# An interpretable machine learning model for biomarker identification and diagnostic nomogram development in hypo-productive thrombocytopenia

**DOI:** 10.1038/s41598-026-58489-9

**Published:** 2026-06-20

**Authors:** Junjie Wang, Baozhi Fang, Peng Wang, Xiao Yu, Yifei Zhou, Muzhi Yuan, Mingen Lyu

**Affiliations:** Department of Hematology, The Affiliated Suzhou Hospital of Nanjing Medical University, Suzhou, China

**Keywords:** Hypo-productive thrombocytopenia, Machine learning, Biomarkers, Diagnostic nomogram, SHAP, Biomarkers, Computational biology and bioinformatics, Diseases, Medical research, Risk factors

## Abstract

Thrombocytopenia is a common hematological disorder with diverse etiologies. This study aimed to identify potential biomarkers and to develop an interpretable diagnostic nomogram for the early recognition of hypo-productive thrombocytopenia. This retrospective study included 185 patients with thrombocytopenia who were admitted to the Department of Hematology at the Affiliated Suzhou Hospital of Nanjing Medical University between 2020 and 2025. Patients were categorized into hypo-productive thrombocytopenia (*n* = 114) and hyper-destructive thrombocytopenia (*n* = 71) according to etiology. Feature selection was performed using least absolute shrinkage and selection operator (LASSO) regression, support vector machine recursive feature elimination (SVM-RFE), and Boruta algorithms, followed by univariate and multivariate logistic regression analyses. Six machine learning (ML) models were developed and compared based on residual analysis and receiver operating characteristic (ROC) curve. The optimal model was interpreted using SHapley Additive exPlanations (SHAP) analysis. The diagnostic nomogram was evaluated by the ROC, calibration curves, and decision curve analysis (DCA). Finally, age, platelet-to-lymphocyte ratio (PLR), neutrophil-to-lymphocyte ratio (NLR), and mean platelet volume (MPV) were identified as key predictors. The generalized linear model(GLM)–based nomogram demonstrated strong discriminative ability (area under the curve [AUC] = 0.945, 95% confidence interval [CI] 0.900–0.976) with excellent calibration. DCA demonstrated a higher net benefit for the nomogram than individual predictors. The interpretable machine learning derived nomogram may serve as a practical and non-invasive tool to assist in the early identification of hypo-productive thrombocytopenia.

## Introduction

Thrombocytopenia is a common and highly heterogeneous disorder characterized by a reduced platelet count in peripheral blood, typically defined as a platelet count < 150 × 10^9^/L^1,2^. According to underlying pathophysiological mechanisms^3–9^, thrombocytopenia can be classified into two major types: hypo-productive thrombocytopenia and hyper-destructive thrombocytopenia^10,11^. Hypo-productive thrombocytopenia primarily results from impaired bone marrow hematopoiesis and is commonly observed in conditions such as aplastic anemia (AA), acute leukemia, and myelodysplastic syndromes (MDS)^12^. In contrast, hyper-destructive thrombocytopenia is caused by excessive peripheral platelet destruction or consumption and is frequently associated with immune thrombocytopenia (ITP), disseminated intravascular coagulation (DIC), thrombotic thrombocytopenic purpura (TTP), autoimmune diseases, pregnancy-related disorders, and mechanical platelet injury^13^. Bone marrow aspiration and biopsy remain the gold standard for evaluating hematopoietic function. However, these invasive procedures are costly and carry risks of bleeding and infection, making them suboptimal as first-line assessments for patients with thrombocytopenia of unclear etiology. Given that different etiologies are associated with markedly different treatment strategies and prognoses, early and accurate identification of hypo-productive thrombocytopenia is of great importance for clinical decision-making.

In recent years, Platelet parameters, including platelet count (PLT), plateletcrit (PCT), platelet large cell ratio (P-LCR), mean platelet volume (MPV), and platelet distribution width (PDW), collectively reflect the balance between platelet production and destruction and may indirectly indicate bone marrow hematopoietic activity. Previous studies have demonstrated that platelet parameters differ significantly among patients with thrombocytopenia of different etiologies, suggesting their potential value for etiological discrimination^14–17^. In addition, derived immune-inflammatory indices have been widely studied in hematological malignancies, such as Hodgkin lymphoma^18^, multiple myeloma^19^, and diffuse large B-cell lymphoma^20^, where they have been found to correlate closely with disease progression and prognosis. A study by de Jong MME et al. demonstrated that inflammatory cytokines impairing the maintenance of normal hematopoietic stem and progenitor cells, ultimately driving bone marrow microenvironment remodeling and hematopoietic dysfunction^21^.

Machine learning algorithms offer clear advantages in handling high-dimensional, multivariate, and nonlinear data, and have been increasingly applied in diagnostic modeling and disease risk prediction^22,23^. In thrombocytopenia research, however, most studies have been limited to specific disease contexts, with relatively few efforts aimed at systematically distinguishing subtypes based on underlying pathophysiological mechanisms. To address this gap, the present study integrates five platelet parameters and eight derived immune-inflammatory indices and applies machine learning approaches to identify key biomarkers associated with hypo-productive thrombocytopenia. An interpretable SHAP-based nomogram model was subsequently developed^24,25^. Leveraging routinely available laboratory data, this noninvasive tool aims to support clinical decision-making and reduce unnecessary traumatic examinations.

## Methods

### Study design

This study was a single-center retrospective cohort study. Participants were patients diagnosed with thrombocytopenia in the Department of Hematology at the Affiliated Suzhou Hospital of Nanjing Medical University. The study protocol was approved by the Ethics Committee of The Affiliated Suzhou Hospital of Nanjing Medical University (Approval No. K-2026-080-K01, Date 20 March 2026). Given the retrospective nature of the study, the requirement for informed consent was waived. All procedures were conducted in accordance with the Declaration of Helsinki.

### Study population

All adult patients diagnosed with thrombocytopenia in the Department of Hematology at the Affiliated Suzhou Hospital of Nanjing Medical University between January 2020 and December 2025 were included in this study. The inclusion criterion was all adult patients with a platelet count < 150 × 10^9^/L. The exclusion criteria were strictly defined as follows: (1) pseudo thrombocytopenia; (2) receipt of blood transfusion or platelet transfusion within 7 days prior to blood cell analysis; (3) current use of antiplatelet agents or other medications known to induce thrombocytopenia; (4) thrombocytopenia secondary to physiological conditions, such as pregnancy; (5) major surgery within the preceding month that could potentially affect platelet levels; (6) thrombocytopenia primarily caused by splenic sequestration; (7) clinically recognized mixed-mechanism thrombocytopenia in which a predominant pathophysiological category could not be reliably determined; (8) severe active infection or systemic inflammatory conditions that could significantly influence hematological inflammatory markers, and (9) patients with missing key clinical or laboratory data.

### Data collection and variable definitions

Demographic characteristics, laboratory results, and final diagnostic outcomes were extracted from the electronic medical record system. Variables with more than 30% missing data were excluded from analysis. Demographic variables included sex, age, and body mass index (BMI). Laboratory results included Absolute Neutrophil Count (ANC), Absolute Lymphocyte Count (ALC), Absolute Monocyte Count (AMC), Red Cell Distribution Width (RDW), Lactate Dehydrogenase (LDH), Albumin (ALB), and Blood Urea Nitrogen (BUN). Platelet parameters included PLT, PCT, P-LCR, MPV, and PDW. Derived immunoinflammatory indices were calculated from routine hematological and biochemical tests, including PLR, NLR, RPR, SII, LAU, LAR, PAR, and PIV. The indices were calculated as follows: BMI = weight/height^2^; PLR = PLT/ALC; NLR = ANC/ALC; RPR = RDW-CV/PLT; SII = (PLT × ANC)/ALC; LAU = (LDH/BUN)/ALB; LAR = LDH/ALB; PAR = PLT/ALB; PIV = (ANC × AMC × PLT)/ALC. All laboratory measurements were obtained from the first test prior to any disease-specific treatment.

### Patient classification and outcome definition

Based on bone marrow examination, laboratory indicators, imaging studies, and final diagnosis, patients were classified into a hypo-productive thrombocytopenia group and a hyper-destructive thrombocytopenia group. Hypo-productive thrombocytopenia was defined as thrombocytopenia primarily resulting from impaired bone marrow hematopoietic function, whereas hyper-destructive thrombocytopenia was defined as thrombocytopenia mainly caused by increased peripheral platelet destruction or consumption. Hypo-productive thrombocytopenia was considered the positive outcome for subsequent analyses. The hypo-productive thrombocytopenia group included AA (*n* = 17), MDS (*n* = 18), leukemia (*n* = 26), MM (*n* = 14), megaloblastic anemia (*n* = 12), and lymphoma (*n* = 27). The hyper-destructive thrombocytopenia group primarily consisted of ITP (*n* = 62) and secondary immune thrombocytopenia (*n* = 9).

### Statistical analysis

All statistical analyses were performed using R software (version 4.4.1; https://www.r-project.org). To ensure reproducibility, a fixed random seed (set.seed = 1000) was applied before data partitioning and model training. The Shapiro–Wilk test was used to assess the normality of continuous variables. Continuous variables with a normal distribution were expressed as mean ± standard deviation (SD), whereas non-normally distributed variables were presented as median and interquartile range (IQR). Categorical variables were summarized as counts and percentages. Between-group comparisons were conducted using the independent-samples t-test or the Mann–Whitney U test for continuous variables, depending on their distribution, and the chi-square test or Fisher’s exact test for categorical variables, as appropriate. All statistical tests were two-sided, and *p* < 0.05 was considered statistically significant.

### Feature variable selection

Feature selection was performed using three machine learning algorithms—LASSO, SVM-RFE, and Boruta—combined with univariate and multivariate logistic regression analyses. LASSO regression was applied to identify candidate predictors by performing tenfold cross-validation to determine the optimal penalty parameter (λ). Variables corresponding to nonzero coefficients at the λ_min criterion were selected as candidate features. SVM-RFE constructs a classification model based on support vector machines, evaluates the contribution of each feature to the classification outcome according to its weight in the model, and recursively removes the feature with the least contribution in each iteration. The classification accuracy of different feature subsets was recorded throughout the recursive process, and the subset with the highest accuracy was selected as candidate features. The Boruta algorithm is a random forest–based feature selection method that creates shadow variables by randomly permuting each original variable. The importance of original variables is then compared with that of shadow variables, and features significantly outperforming shadow variables are selected as candidate predictors according to the Boruta decision rules. The final candidate features were defined as the intersection of variables selected by LASSO, SVM-RFE, and Boruta. These features were subsequently included in univariate logistic regression analyses, and variables with *p* < 0.05 were further incorporated into multivariate logistic regression to identify the core biomarkers associated with hypo-productive thrombocytopenia.

### Model development, evaluation and interpretation

The dataset was divided into a training cohort and a test cohort at a 7:3 ratio using stratified random sampling implemented through the caret package, thereby preserving the class distribution between cohorts, with the training set used for model development and the test set for validation. Six machine learning models were constructed in R, including the Generalized Linear Model (GLM), k-Nearest Neighbor (KNN), Least Absolute Shrinkage and Selection Operator (LASSO) regression, Support Vector Machine (SVM), Gradient Boosting Machine (GBM), and Decision Tree (DT). All machine learning models were developed within the caret framework using tenfold cross-validation for internal resampling and model optimization. Model performance was evaluated by examining residual distributions and calculating the root mean square error (RMSE) to assess overall model fit. Discriminative ability was assessed using ROC curves and the corresponding AUC. Considering predictive performance, stability, and interpretability, the optimal model was selected for further analysis. Hyperparameter optimization was performed using the tuning procedures implemented within the caret framework where applicable. For the LASSO model, a predefined tuning grid was applied with alpha fixed at 1 and lambda values ranging from 0.01 to 1. The remaining models were trained using the default optimization settings provided by the corresponding R packages.

SHAP analysis was employed to provide visualization of feature contributions at both the population and individual levels, thereby enhancing clinical interpretability and transparency of model predictions. Based on the final set of selected key biomarkers, a diagnostic nomogram was constructed to predict individual risk of hypo-productive thrombocytopenia. The discriminative ability of the nomogram was assessed using ROC curves, and the optimal probability threshold for diagnosis was determined using the Youden index. Calibration of the model was evaluated with calibration curves, and clinical utility was assessed using DCA.

## Results

### Baseline characteristics of study population

**Fig. 1:**
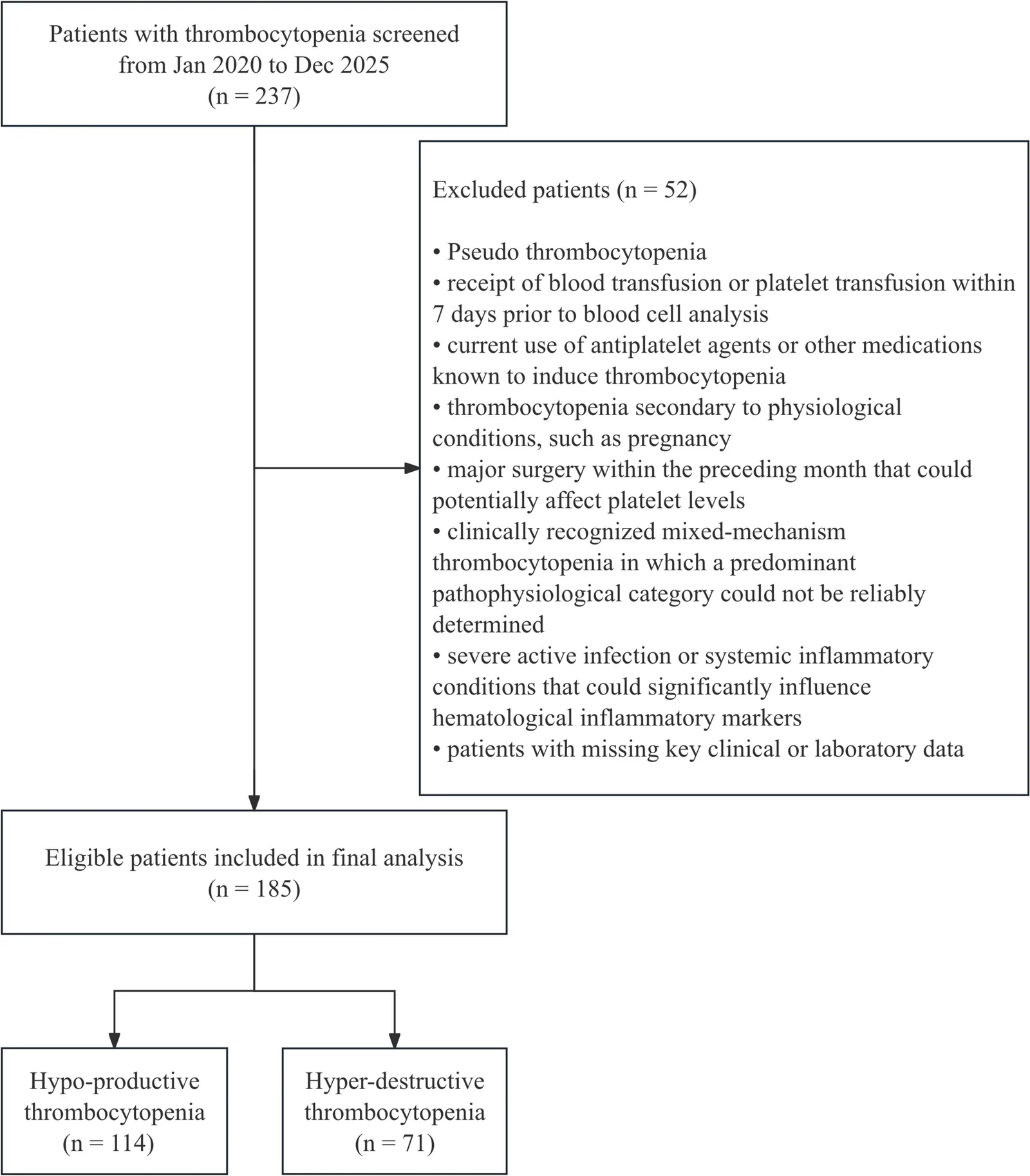
Flowchart of patient selection.

A total of 237 patients with thrombocytopenia were initially screened. After applying the predefined inclusion and exclusion criteria, 185 eligible patients were ultimately included in the final analysis, including 114 patients with hypo-productive thrombocytopenia and 71 patients with hyper-destructive thrombocytopenia. The patient selection process is illustrated in Fig. 1. The hypo-productive group was predominantly male, whereas the hyper-destructive group had a higher proportion of females (*p* < 0.001). The median age of patients in the hyper-destructive group was 51 years, which was lower than that of the hypo-productive group (*p* < 0.001). No significant difference in BMI was observed between the two groups.

Among platelet parameters, all indices except PCT showed statistically significant differences (*p* < 0.001). Regarding the derived immune-inflammatory indices, all except RPR and LAU were significantly different between the two groups (*p* < 0.05), although the standardized mean differences (SMD) were all less than 1 (Table 1).

**Table 1:** Baseline characteristics of hypo-productive and hyper-destructive.

Variables	Hypo-productive (*n* = 114)	Hyper-destructive (*n* = 71)	*p*-value	SMD
Sex = 0/1	48/66 (42.1/57.9)	44/27 (62.0/38.0)	0.013	0.406
Age	69.50 [61.25, 75.00]	51.00 [32.00, 63.50]	< 0.001	0.947
BMI (kg/m²)	23.28 [20.89, 25.65]	23.56 [20.23, 26.32]	0.665	0.096
PLT (×10^9^/L)	58.00 [31.00, 84.00]	33.00 [23.50, 54.00]	< 0.001	0.555
PCT (%)	0.06 [0.03, 0.09]	0.04 [0.03, 0.07]	0.101	0.229
P-LCR (%)	31.30 [23.65, 37.10]	46.60 [39.45, 55.60]	< 0.001	1.715
MPV (fL)	10.65 [9.72, 11.60]	12.90 [12.40, 13.95]	< 0.001	1.740
PDW (%)	15.15 [12.20, 16.17]	16.50 [15.60, 17.70]	< 0.001	1.108
PLR	47.80 [19.17, 104.05]	26.36 [12.95, 55.64]	0.002	0.597
RPR	0.27 [0.18, 0.55]	0.39 [0.24, 0.59]	0.072	0.042
NLR	1.41 [0.51, 2.38]	2.95 [1.75, 6.04]	< 0.001	0.707
SII	63.31 [15.96, 182.00]	113.26 [61.36, 239.68]	0.001	0.179
LAU	1.06 [0.65, 2.10]	0.87 [0.73, 1.11]	0.091	0.365
LAR	5.49 [4.79, 9.43]	4.66 [4.01, 5.51]	< 0.001	0.405
PAR	1.64 (0.98)	1.07 (0.71)	< 0.001	0.662
PIV	14.22 [3.27, 66.38]	36.47 [20.85, 110.30]	< 0.001	0.176

Continuous variables are presented as mean ± standard deviation or medians and inter-quartile spacing and categorical variables are expressed as percentages.

Abbreviations: *BMI* body mass index, *PLR* platelet lymphocyte ratio, *RPR* red blood cell distribution width-platelet ratio, *NLR* neutrophil lymphocyte ratio, *SII* systemic immune inflammatory index, *LAU* lactate dehydrogenase/albumin to urea ratio, *LAR* lymphocyte albumin ratio, *PAR* platelet albumin ratio, *PIV* pan-immune-inflammation value.

### Features selected in models

**Fig. 2:**
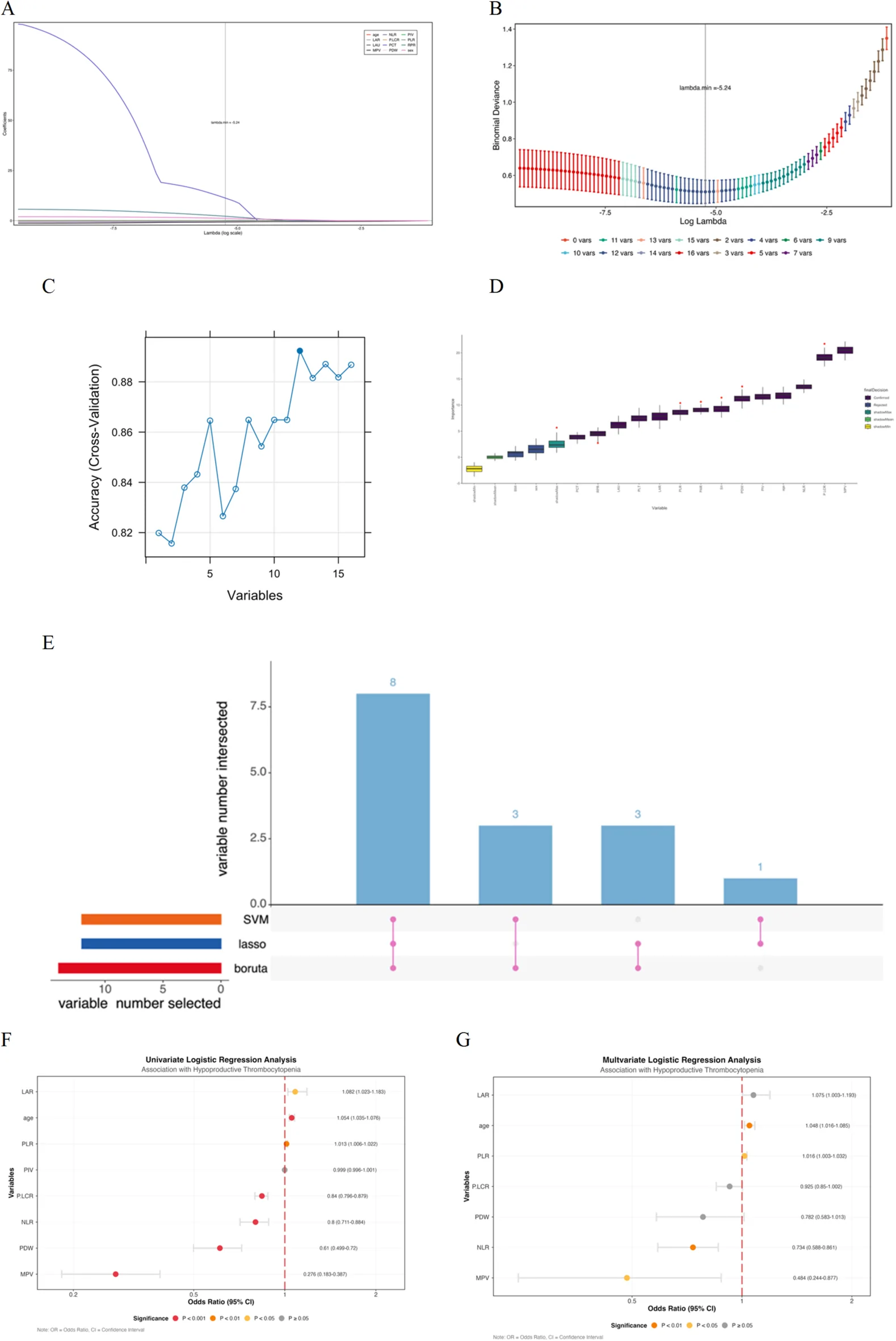
Screening of core variables. (**A**) Coefficient paths for LASSO regression when lambda.min=-5.24. (**B**) Cross-validation plot for LASSO regression when lambda.min=-5.24. (**C**) The Cross-Validation accuracy curve assists in identifying the optimal number of features by evaluating predictive stability across different subsets. (**D**) Visualization of the results of the BORUTA feature selection algorithm. Boxplots illustrate the distribution of feature importance scores across multiple iterations. (**E**) UpSet diagram of the model filtering variables. Single pink dots represent variables uniquely selected by a specific model, while connected pink dots indicate variables shared across multiple models. (**F**) Univariate analysis of factors associated with Hypo-productive Thrombocytopenia. (**G**) Multivariate analysis of factors associated with Hypo-productive Thrombocytopenia. The color of the circles reflects the level of statistical significance.

The 16 candidate variables were screened using three machine learning algorithms—LASSO, SVM-RFE, and Boruta—based on the outcome variable, as shown in Fig. 2A and B. LASSO regression identified 12 variables. SVM-RFE selected 12 variables (Fig. 2C). Boruta identified 14 variables (Fig. 2D). An UpSet plot demonstrated the 8 variables shared across all three methods: age, P-LCR, MPV, PDW, PLR, NLR, LAR, and PIV (Fig. 2E).

Univariate logistic regression analysis indicated that all variables except PIV were significantly associated with hypo-productive thrombocytopenia (Fig. 2F). Variables with statistical significance in the univariate analysis were subsequently included in multivariate logistic regression. The final core predictors were age (Odds Ratio [OR], 1.048; 95% CI, 1.016–1.085), PLR (OR, 1.016; 95% CI, 1.003–1.032), NLR (OR, 0.734; 95% CI, 0.588–0.861), and MPV (OR, 0.484; 95% CI, 0.244–0.877) (Fig. 2G).

### Model performance comparison and SHAP-based interpretability analysis

**Fig. 3:**
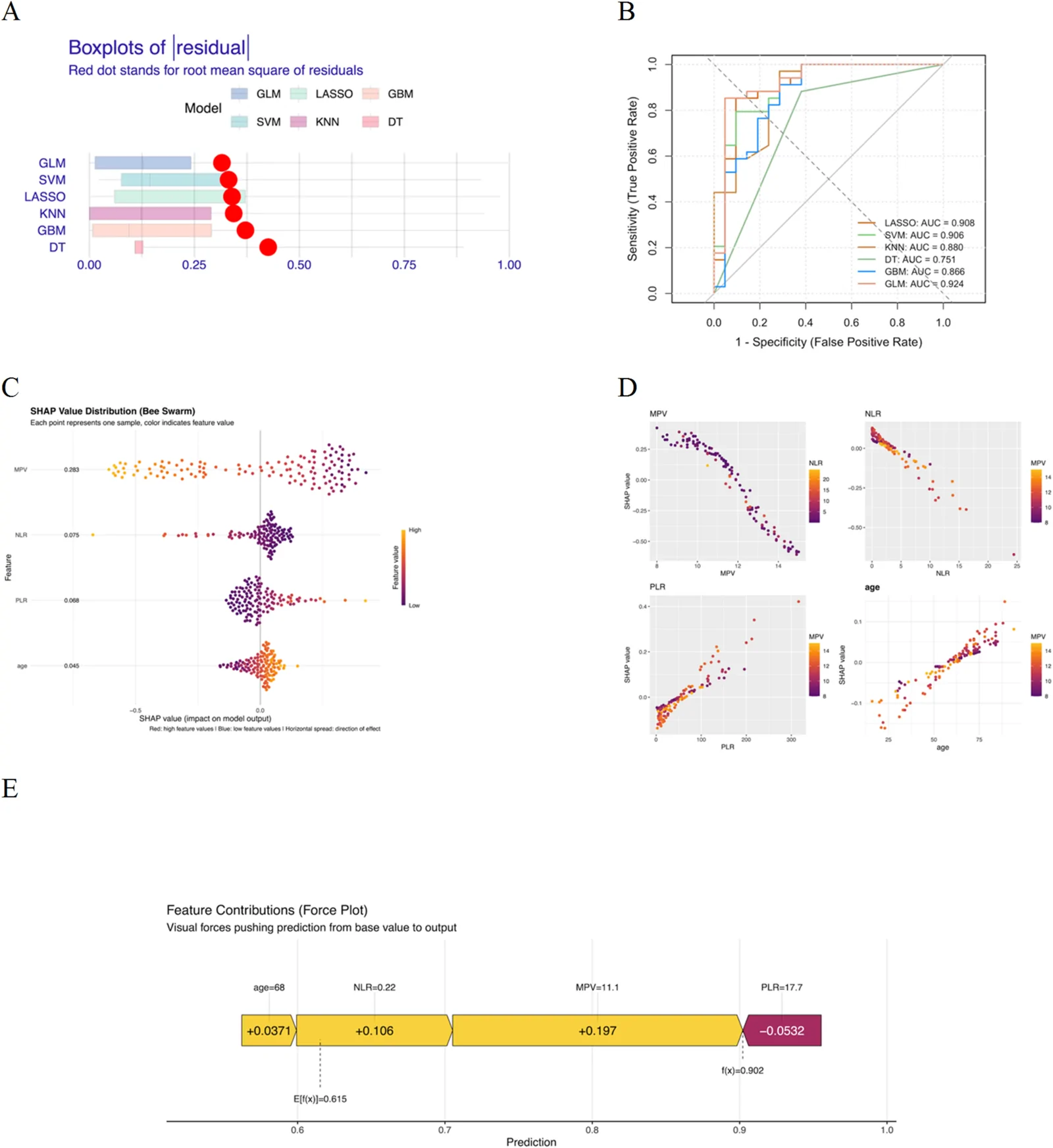
Assessment of the predictive power of thrombocytopenia by core variables. (**A**) Boxplots of absolute residual values for different models in the validation set. The red dots represent the RMSE of each model. (**B**) Comparison of ROC curves of six models in the validation set. (**C**) Beeswarm plot of SHAP values for core variables. (**D**) SHAP feature dependence scatter plot. These plots illustrate potential nonlinear associations between features and model output, as well as possible interaction effects. (**E**) SHAP force plot for a representative individual case. Yellow segments indicate positive contributions, whereas the purple-red segment indicates a negative contribution.

In the training set, age, PLR, NLR, and MPV were used as predictors, with hypo-productive thrombocytopenia as the outcome variable, to construct six machine learning models. Based on the absolute residuals and ROC curves of each model, the optimal model was selected for subsequent analyses. The GLM model demonstrated the smallest residuals and the highest AUC = 0.924 in the validation set (Fig. 3A and B), indicating superior predictive performance for hypo-productive thrombocytopenia.

SHAP analysis was subsequently employed to quantify the contribution of each core predictor to the GLM model. The beeswarm plot illustrated the distribution of feature effects, with features ordered by mean absolute SHAP value. MPV contributed the most (|SHAP| = 0.283), followed by NLR, PLR, and age (Fig. 3C). SHAP dependence plots showed that increasing levels of MPV and NLR were associated with higher contributions to model predictions, whereas increasing PLR and age were associated with lower contributions. No significant interactions among the features were observed (Fig. 3D). A waterfall plot further illustrated the impact of individual features on the prediction for a single patient (Fig. 3E).

### Nomogram construction and validation

**Fig. 4:**
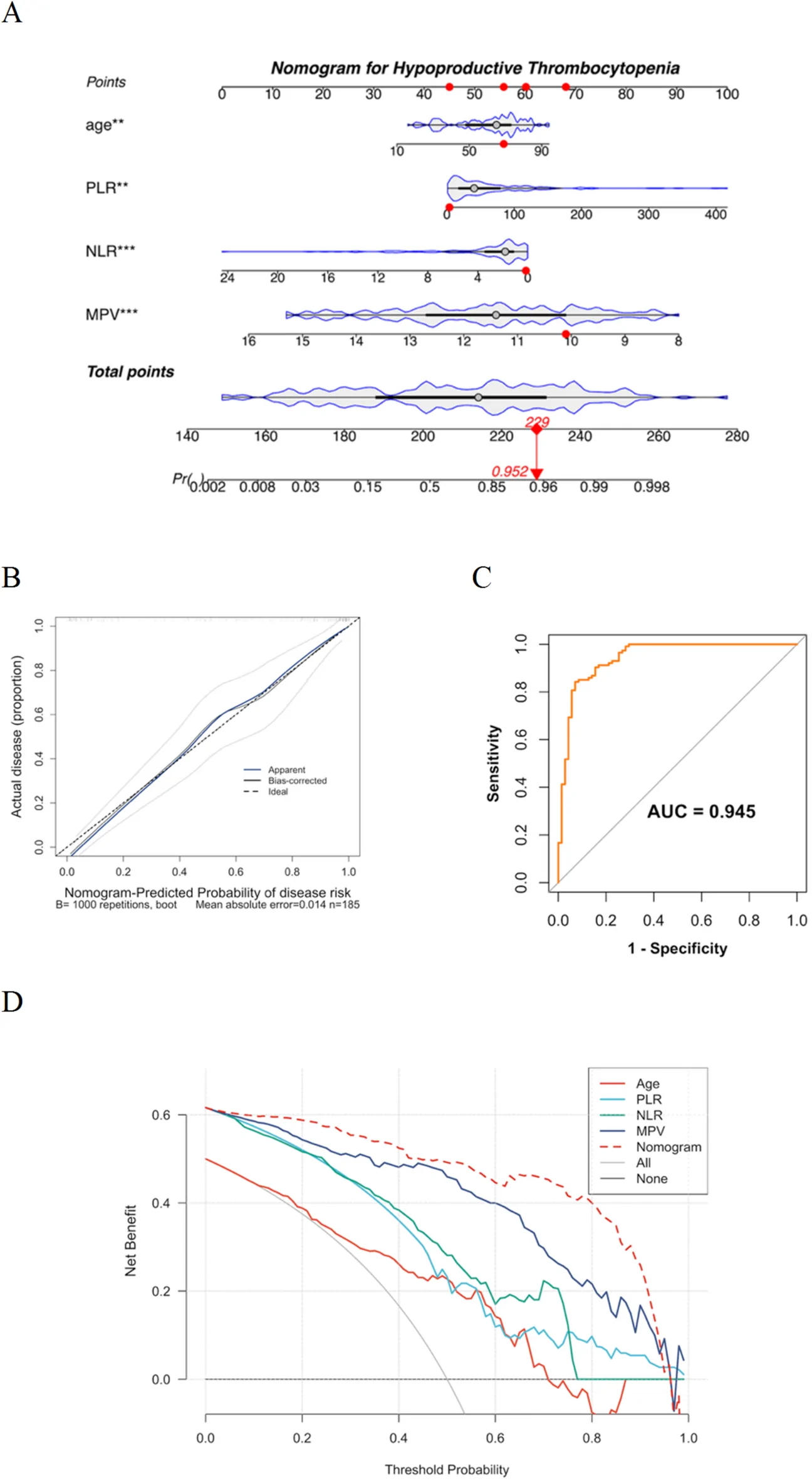
Development and validation of nomogram model to predict Hypo-productive thrombocytopenia. (**A**) Nomogram for the diagnosis of Hypo-productive thrombocytopenia. (**B**) Calibration curve for the nomogram model (1,000 resamples). (**C**) ROC curve of the nomogram. (**D**) Decision curves for the nomogram and individual predictors (age, PLR, NLR, MPV).

The nomogram incorporating age, PLR, NLR, and MPV demonstrated good predictive performance for hypo-productive thrombocytopenia (Fig. 4A). Calibration curves indicated high agreement between predicted and observed probabilities (RMSE = 0.014) (Fig. 4B). ROC curve analysis showed excellent discriminative ability, with an AUC of 0.945 (95% CI, 0.900–0.976) (Fig. 4C). Based on the maximum Youden index of 0.772, the optimal probability threshold was 0.793, corresponding to a sensitivity of 0.842 and a specificity of 0.930.

Decision curve analysis revealed that at a threshold probability of 0.2, the nomogram provided a net benefit of 0.588, which was higher than that of any individual predictor (age, 0.388; PLR, 0.520; NLR, 0.516; MPV, 0.543), indicating substantial clinical utility (Fig. 4D).

## Discussion

The etiology of thrombocytopenia is complex and diverse and accurately identifying the potential cause and selecting appropriate diagnostic tests during a patient’s initial visit remains a significant challenge for outpatient physicians. This study, from a pathophysiological perspective, combined multiple feature screening algorithms to identify four core predictive factors: age, PLR, NLR, and MPV. Among several machine learning models, the GLM demonstrated the best predictive performance and good residual stability for hypo-productive thrombocytopenia. The diagnostic nomogram exhibited excellent discriminative power (AUC = 0.945), good calibration, and high clinical applicability. Furthermore, to quantify the contribution of each predictive factor and enhance the model’s interpretability, this study conducted a SHAP analysis. The results showed that MPV was the most influential variable, followed by NLR, PLR, and age.

With increasing age, the bone marrow’s self-renewal capacity declines, lineage bias increases, and the microenvironment changes, thereby increasing susceptibility to bone marrow failure syndrome and clonal blood disorders, and the risk of developing hypo-productive thrombocytopenia also increases^26–28^. In contrast, idiopathic thrombocytopenic purpura (ITP), a typical destructive thrombocytopenic disease, is more frequently observed in females due to sex-related immune differences and shows a bimodal age distribution with peaks in childhood and young adulthood^29^. A prospective study also found similar results^30^.

As a key indicator, MPV directly reflects platelet production status. Given that immature platelets are larger than mature ones^31,32^, decreased MPV typically indicates impaired platelet production^33^. In contrast, elevated MPV reflects increased peripheral platelet destruction or consumption with relatively preserved or compensatorily enhanced megakaryopoiesis^13,34,35^. Walle et al. verified that MPV had a significantly higher pooled mean in ITP patients than in hypo-productive thrombocytopenia cases [Weighted Mean Difference (WMD) = 2.03 fl., 95% CI: 1.38–2.69] with an Summary Receiver Operating Characteristic(SROC)-AUC of 0.87^33^, validating MPV as a valuable non-invasive marker for differentiating hyper-destructive and hypo-productive thrombocytopenia. In addition, P-LCR and PDW, which reflect the proportion of large platelets and the heterogeneity of platelet size distribution, can indirectly indicate platelet production dynamics and turnover. In our cohort, both P-LCR and PDW were significantly elevated in the hyper-destructive group (*p* < 0.001), consistent with previous findings^30^. Although these parameters were identified during machine learning-based feature screening, they were excluded from the final multivariable logistic regression model, suggesting that MPV may provide superior robustness and representativeness as an independent predictor.

Inflammation is a key driver of bone marrow microenvironment remodeling and hematopoietic dysfunction^36^. As derived immune-inflammatory indices, NLR and PLR reflect systemic inflammatory status and immune balance^37^. Previous studies have shown that, in hematologic malignancies characterized by ineffective hematopoiesis, persistent inflammation disrupts hematopoietic stem and progenitor cell function and remodels the bone marrow niche through interactions with stromal and immune components, ultimately creating a suppressive microenvironment. Moreover, inflammation-induced immune dysregulation promotes myeloid expansion and lymphocyte suppression, further aggravating hematopoietic impairment^38^. In this study, NLR and PLR were identified as core predictors of hypo-productive thrombocytopenia, highlighting the clinical utility of peripheral inflammation-related markers in recognizing impaired hematopoiesis.

Methodologically, compared with previous studies that often relied on a single machine learning algorithm or simple statistical selection, this study integrated multiple feature selection algorithms with traditional statistical analyses for cross-validation, reducing potential bias and enhancing the robustness and generalizability of variable selection^39–41^. In model selection, rather than relying on complex black-box algorithms, multiple machine learning models, including linear models, were systematically compared. The GLM emerged as the optimal predictive model, suggesting that in clinical studies with relatively limited sample sizes and biologically interpretable variables, simpler and interpretable models may outperform more complex black-box approaches^23,42^. Although the GLM is inherently interpretable, SHAP analysis further enhanced model transparency by enabling visualization of feature contributions at both global and individual levels. Compared with conventional odds ratios, SHAP values provide more intuitive decomposition of prediction outputs and facilitate clinical interpretation of individualized risk estimation^43–45^. The nomogram further visualized the model, facilitating translation into clinical practice.

Nevertheless, several limitations should be acknowledged. First, derived inflammatory indices are vulnerable to confounding factors, including infection, chronic inflammatory conditions, and medications, despite strict exclusion criteria, residual confounding cannot be fully ruled out due to the retrospective design. Second, the exclusion of splenic sequestration and mixed etiologies may oversimplify the pathophysiological heterogeneity and limit real-world generalizability. Finally, as a single-center retrospective study, external validation in large, multicenter prospective cohorts is warranted to confirm the robustness and applicability of the proposed model.

## Conclusion

This study focused on the clinical decision-making needs of patients with newly diagnosed thrombocytopenia of unknown etiology and evaluated the utility of platelet parameters and derived immune-inflammatory indices for etiological differentiation. The GLM-based diagnostic nomogram demonstrated good discrimination, stability, and interpretability, providing a noninvasive and convenient tool for the early identification of hypo-productive thrombocytopenia.

## Data Availability

The datasets generated and/or analyzed during the current study available from the corresponding author on reasonable request.

## Abbreviations

AA: Aplastic anemia
ALB: Albumin
ALC: Absolute lymphocyte count
AMC: Absolute monocyte count
ANC: Absolute neutrophil count
AUC: Area under the curve
BMI: Body mass index
BUN: Blood urea nitrogen
CI: Confidence interval
DCA: Decision curve analysis
DIC: Disseminated intravascular coagulation
DT: Decision tree
GBM: Gradient boosting machine
GLM: Generalized linear model
IQR: Interquartile range
ITP: Immune thrombocytopenia
KNN: k-nearest neighbors
LASSO: Least absolute shrinkage and selection operator
LDH: Lactate dehydrogenase
MDS: Myelodysplastic syndromes
ML: Machine learning
MPV: Mean platelet volume
OR: Odds ratio
PCT: Plateletcrit
PDW: Platelet distribution width
P-LCR: Platelet large cell ratio
PLT: Platelet count
RDW: Red cell distribution width
RMSE: Root mean square error
ROC: Receiver operating characteristic
SD: Standard deviation
SHAP: SHapley additive explanations
SMD: Standardized mean differences
SROC: Summary receiver operating characteristic
SVM: Support vector machine
SVM-RFE: Support vector machine recursive feature elimination
TTP: Thrombotic thrombocytopenic purpura
WMD: Weighted mean difference
λ: Optimal penalty parameter

## References

[1] Gauer, R. L. & Thrombocytopenia Evaluation and Management. *J. Am. Fam Physician*. **106**, 288–298 (2022). 36126009

[2] Gremmel, T. & Frelinger, A. L. 3rd&Michelson, A.D. Platelet Physiology. *J. Semin Thromb. Hemost.* **50**, 1173–1186 (2024). 10.1055/s-0044-1786387 38653463

[3] Li, J. & Sullivan, J. A. Pathophysiology of immune thrombocytopenia. *J. Curr. Opin. Hematol.* **25**, 373–381 (2018). 10.1097/MOH.0000000000000447 30015642

[4] Houwerzijl, E. J., Blom, N. R., Van Der Want, J. J., Vellenga, E. & De Wolf, J. T. Megakaryocytic dysfunction in myelodysplastic syndromes and idiopathic thrombocytopenic purpura is in part due to different forms of cell death. *J. Leuk.* **20**, 1937–1942 (2006). 10.1038/sj.leu.2404385 16990774

[5] Chen, X. & Shukla, M. An unconquered challenge in MDS: review of pathophysiology, clinical manifestations, and management options of MDS with thrombocytopenia. *J. Ann. Hematol.* **104**, 4319–4332 (2025). 10.1007/s00277-025-06374-2 PMC12552404 40935929

[6] Singh, A., Uzun, G. & Bakchoul, T. Primary immune thrombocytopenia: Novel insights into pathophysiology and disease management. *J. Clin. Med.* **10**, (2021). 10.3390/jcm10040789 PMC7920457 33669423

[7] Provan, D. Recent advances in the mechanisms and treatment of immune thrombocytopenia. *J. EBioMedicine*. **76**, 103820 (2022). 10.1016/j.ebiom.2022.103820 PMC8792416 35074629

[8] Chen, Y. et al. Association of Platelet Desialylation and Circulating Follicular Helper T Cells in Patients With Thrombocytopenia. *J. Front. Immunol.* **13**, 810620 (2022). 10.3389/fimmu.2022.810620 PMC9016750 35450072

[9] Townsley, D. M., Desmond, R. & Dunbar, C. E. Pathophysiology and management of thrombocytopenia in bone marrow failure: possible clinical applications of TPO receptor agonists in aplastic anemia and myelodysplastic syndromes. *J. Int. J. Hematol.* **98**, 48–55 (2013). 23690288 10.1007/s12185-013-1352-6 PMC4144663

[10] Stasi, R. How to approach thrombocytopenia. *J. Hematol. Am. Soc. Hematol. Educ. Program*. 191–197 (2012). 10.1182/asheducation-2012.1.191 23233580

[11] Numbenjapon, T. et al. A prospective evaluation of normal mean platelet volume in discriminating hyperdestructive thrombocytopenia from hypoproductive thrombocytopenia. *J. Int. J. Lab. Hematol.* **30**, 408–414 (2008). 18522712 10.1111/j.1751-553X.2007.00969.x

[12] Asghar, M. B. et al. Diagnostic Accuracy of Immature Platelet Fraction (IPF) to Differentiate Between Thrombocytopenia due to Peripheral Destruction < em>versus Bone Marrow Failure. *J. J. Coll. Physicians Surg. Pak*. **33**, 760–764 (2023). 37401216 10.29271/jcpsp.2023.07.760

[13] Jesudas, R. Where have all the platelets gone? HIT, DIC, or something else? *J. Hematol. Am. Soc. Hematol. Educ. Program*. 43–50 (2023). 10.1182/hematology.2023000465 PMC10727081 38066886

[14] Chen, W. et al. Platelet parameters for distinguishing between inherited macrothrombocytopenia and acquired thrombocytopenia: a retrospective case-control study. *J. Platelets*. **36**, 2489025 (2025). 10.1080/09537104.2025.2489025 40211875

[15] Senthil Nathan, S., Varadaraj, P. & Nallusamy, G. The Significance of Platelet Indices in the Evaluation of Thrombocytopenia. *J. Cureus*. **16**, e65756 (2024). 10.7759/cureus.65756 PMC11361324 39211710

[16] Wang, W. et al. The Value of Combined Detection of Megakaryocyte and Platelet Parameters for the Diagnosis of Primary Immune Thrombocytopenia. *J. Clin. Appl. Thromb. Hemost.* **28**, 10760296221106779 (2022). 10.1177/10760296221106779 PMC9358600 35924375

[17] Adly, A. A., Ragab, I. A. & Ismail, E. A. Evaluation of the immature platelet fraction in the diagnosis and prognosis of childhood immune thrombocytopenia. *J. Platelets*. **26**, 645–650 (2015). 10.3109/09537104.2014.969220 25350586

[18] Reddy, J. P. et al. Pre-treatment neutrophil/lymphocyte ratio and platelet/lymphocyte ratio are prognostic of progression in early stage classical Hodgkin lymphoma. *J. Br. J. Haematol.* **180**, 545–549 (2018). 29271057 10.1111/bjh.15054

[19] Sun, Y. Q., Li, Q. F., Zhang, Q. K. & Wei, X. F. [Significance of Neutrophil/Lymphocyte Ratio in the Prognosis of Patients with Multiple Myeloma]. *J. Zhongguo Shi Yan Xue Ye Xue Za Zhi*. **27**, 489–493 (2019). 10.19746/j.cnki.issn.1009-2137.2019.02.029 30998159

[20] Liu, J., Zhang, S., Mi, R. & Chen, L. Prognostic significance of the neutrophil-to-lymphocyte ratio in peripheral T-cell lymphoma: a meta-analysis. *J. Cancer Cell. Int.* **21**, 688 (2021). 10.1186/s12935-021-02391-z PMC8684640 34923981

[21] De Jong, M. M. E., Chen, L. & Raaijmakers, M. &Cupedo, T. Bone marrow inflammation in haematological malignancies. *J. Nat. Rev. Immunol.* **24**, 543–558 (2024). 10.1038/s41577-024-01003-x 38491073

[22] Fu, Y. & Zhao, J. LASSO regression and Boruta algorithm to explore the relationship between neutrophil percentage to albumin ratio and asthma: results from the NHANES 2001 to 2018. *J. Clin. Exp. Med.* **25**, 149 (2025). 10.1007/s10238-025-01701-3 PMC12065745 40347409

[23] Dong, W. et al. Interpretable machine learning analysis of immunoinflammatory biomarkers for predicting CHD among NAFLD patients. *J. Cardiovasc. Diabetol.* **24**, 263 (2025). 40611098 10.1186/s12933-025-02818-1 PMC12224351

[24] Tourab, H. et al. The Use of Machine Learning and Explainable Artificial Intelligence in Gut Microbiome Research: A Scoping Review. *J. IEEE J. Biomed. Health Inf.* **30**, 717–731 (2026). 10.1109/JBHI.2025.3593198 41066278

[25] Ponce-Bobadilla, A. V., Schmitt, V., Maier, C. S. & Mensing, S. Practical guide to SHAP analysis: Explaining supervised machine learning model predictions in drug development. *J. Clin. Transl Sci.* **17**, e70056 (2024). 10.1111/cts.70056 PMC11513550 39463176

[26] De Haan, G. Aging of hematopoietic stem cells. *J. Blood*. **131**, 479–487 (2018). 10.1182/blood-2017-06-746412 29141947

[27] Pang, W. W. et al. Human bone marrow hematopoietic stem cells are increased in frequency and myeloid-biased with age. *J. Proc. Natl. Acad. Sci. U S A*. **108**, 20012–20017 (2011). 22123971 10.1073/pnas.1116110108 PMC3250139

[28] Winter, S. et al. Clonal hematopoiesis and its impact on the aging osteo-hematopoietic niche. *J. Leuk.* **38**, 936–946 (2024). 10.1038/s41375-024-02226-6 PMC11073997 38514772

[29] Nohria, V. & Immune Thrombocytopenia *J. N Engl. J. Med.* **382**, 1077 (2020). 32160680 10.1056/NEJMc1913324

[30] Saran, K., Vidya, K., Seema, K. & Prasad, A. Study of platelet indices and their role in evaluation of thrombocytopenia. *J. J. Family Med. Prim. Care*. **11**, 6236–6242 (2022). 36618137 10.4103/jfmpc.jfmpc_460_22 PMC9810959

[31] Reusswig, F. & An, O. Platelet life cycle during aging: function, production and clearance. *J. Platelets*. **35**, 2433750 (2024). 10.1080/09537104.2024.2433750 39618096

[32] An, O. Platelet lifespan and mechanisms for clearance. *J. Curr. Opin. Hematol.* **31**, 6–15 (2024). 10.1097/MOH.0000000000000792 37905750

[33] Walle, M., Arkew, M., Asmerom, H. & Tesfaye, A. The diagnostic accuracy of mean platelet volume in differentiating immune thrombocytopenic purpura from hypo-productive thrombocytopenia: A systematic review and meta-analysis. *J. PLoS One*. **18**, e0295011 (2023). 10.1371/journal.pone.0295011 PMC10688894 38033118

[34] Kaito, K. et al. Platelet size deviation width, platelet large cell ratio, and mean platelet volume have sufficient sensitivity and specificity in the diagnosis of immune thrombocytopenia. *J. Br. J. Haematol.* **128**, 698–702 (2005). 15725092 10.1111/j.1365-2141.2004.05357.x

[35] Audia, S., Mahévas, M., Samson, M. & Godeau, B. &Bonnotte, B. Pathogenesis of immune thrombocytopenia. *J. Autoimmun. Rev.* **16**, 620–632 (2017). 10.1016/j.autrev.2017.04.012 28428120

[36] Méndez-Ferrer, S. et al. Bone marrow niches in haematological malignancies. *J. Nat. Rev. Cancer*. **20**, 285–298 (2020). 10.1038/s41568-020-0245-2 PMC9912977 32112045

[37] Li, K., Xiang, J. & Li, Y. Neutrophil-to-Lymphocyte Ratio and Platelet-to-Lymphocyte Ratio in Differentiating Appendicitis Severity: A Meta-analysis. *J. J. Surg. Res.* **317**, 365–379 (2026). 41406544 10.1016/j.jss.2025.11.040

[38] Bahmani, F. et al. Bone marrow microenvironment in myelodysplastic neoplasms: insights into pathogenesis, biomarkers, and therapeutic targets. *J. Cancer Cell. Int.* **25**, 175 (2025). 10.1186/s12935-025-03793-z PMC12065391 40349084

[39] Yang, Y. L. et al. Development and validation of a nutrition-integrated nomogram for predicting 28-day mortality in sepsis patients. *J. Front. Nutr.* **12**, 1726151 (2025). 10.3389/fnut.2025.1726151 PMC12864124 41640744

[40] Wan, R. et al. Screening for depression risk in asthma patients: Development and external validation of a machine learning-based predictive model. *J. J. Affect. Disord*. **394**, 120520 (2026). 41151724 10.1016/j.jad.2025.120520

[41] Huang, J. et al. Machine learning prediction of suicide attempts in major depression: Feature selection and model development using national survey data. *J. J. Affect. Disord*. **394**, 120610 (2026). 41197916 10.1016/j.jad.2025.120610

[42] Cai, Z. R. et al. Construction of exosome non-coding RNA feature for non-invasive, early detection of gastric cancer patients by machine learning: a multi-cohort study. *J. Gut*. **74**, 884–893 (2025). 10.1136/gutjnl-2024-333522 PMC12229063 39753334

[43] Liang, Q., Wang, J., Nong, Q. & Tao, S. Integration of multi-omics and machine learning strategies identifies immune related candidate biomarkers in inflammation-associated hypertrophic cardiomyopathy. *J. Front. Immunol.* **16**, 1645382 (2025). 10.3389/fimmu.2025.1645382 PMC12510942 41080564

[44] Bai, X. et al. Identifying liver cirrhosis in patients with chronic hepatitis B: an interpretable machine learning algorithm based on LSM. *J. Ann. Med.* **57**, 2477294 (2025). 10.1080/07853890.2025.2477294 PMC11924261 40104981

[45] Tang, T. et al. Interpretable machine learning model for predicting post-hepatectomy liver failure in hepatocellular carcinoma. *J. Sci. Rep.* **15**, 15469 (2025). 10.1038/s41598-025-97878-4 PMC12048636 40316613

